# No synergistic effect of fecal microbiota transplantation and shugan decoction in water avoidance stress-induced IBS-D rat model

**DOI:** 10.3389/fmicb.2022.995567

**Published:** 2022-09-12

**Authors:** Yangyang Meng, Ya Feng, Lu Hang, Yan Zhou, Enkang Wang, Jianye Yuan

**Affiliations:** Institute of Digestive Diseases, Longhua Hospital, Shanghai University of Traditional Chinese Medicine, Shanghai, China

**Keywords:** water avoidance stress, irritable bowel syndrome, visceral hypersensitivity, fecal microbiota transplantation, serotonin

## Abstract

**Background:**

It has been reported that 5-hydroxytryptamine (5-HT, serotonin) metabolism is involved in the pathogenesis of irritable bowel syndrome (IBS) and that either Shugan decoction (SGD) or fecal microbiota transplantation (FMT) can alleviate the symptoms of IBS in patients and animal models. But the synergistic effect of FMT and SGD on 5-HT metabolism and IBS symptoms has not been investigated.

**Aim:**

The main purpose of this study is to observe the synergistic effect of FMT with SGD on symptoms and 5-HT metabolism in IBS-D rats induced by water avoidance stress (WAS). Moreover, the possible material basis of the FMT was investigated.

**Methods:**

In experiment I, rats were randomly divided into seven groups. Control group: routine feeding; WAS→ Control group: routine feeding with fecal microbiota liquid (FML) 1 (derived from rats in WAS group) gavage since the fourth day; WAS group: 10 days WAS with routine feeding; SGD group: 10 days WAS with SGD gavage since the fourth day on the base of routine feeding; Control→ WAS group: 10 days WAS with FML2 (derived from rats in Control group) gavage since the fourth day with routine feeding; SGD→ WAS group: 10 days WAS with FML3 (derived from rats in SGD group) gavage since the fourth day with routine feeding; SGD + (Control→ WAS) group: 10 days WAS with SGD and FML2 (derived from rats in Control group) gavage since the fourth day with routine feeding. In experiment II, rats were randomly divided into three groups. Control group: routine feeding; Control→ WAS group: 10 days WAS with FML2 gavage since the fourth day with routine feeding; FControl→ WAS group: 10 days WAS with FML2 filtrate gavage since the fourth day. The number of fecal pellets output (FPT) and the pain pressure threshold (PPT) were recorded. The histological changes in colon mucosa were observed by hematoxylin-eosin (HE) stain. The number of enterochromaffin cells (ECs), the content of 5-HT, and the expression of serotonin reuptake transporter (SERT) protein in the colon were measured by immunofluorescence or western blotting.

**Results:**

Compared with that in the control group, the PPT and the expression of SERT in the WAS group and that in the WAS→ Control group were decreased with the increased number of ECs and the level of 5-HT in colon. But the FPT was not increased in the WAS→ Control group although that was increased in the WAS group. Compared with that in the WAS group, the FPT, the PPT, the number of ECs, the level of 5-HT, and the expression of SERT protein in colon in the SGD group, control→ WAS group, SGD→ WAS group, and SGD+(Control→ WAS) group were all recovered. The recovery of these indicators in the Control→ WAS group and that in the FControl→ WAS group was not significantly different.

**Conclusion:**

No synergistic effect of SGD with FMT on IBS symptoms induced by WAS was found. The metabolites of intestinal microbiota may be the main active substances of the FML derived from normal rats to alleviate WAS-induced IBS symptoms.

## Introduction

Irritable bowel syndrome (IBS) is a chronic and recurrent functional intestinal disease with abdominal pain, abdominal distension, or abdominal discomfort as the main symptoms, which is related to or accompanied by defecation habits changes without organic lesions (Lacy et al., 2021). According to the Rome IV standard, IBS can be divided into four subtypes: constipation-predominant IBS (IBS-C), diarrhea-predominant IBS (IBS-D), mixed IBS (IBS-M), and unclassified IBS (IBS-U). Among them, IBS-D is identified as the most common subtype of IBS. It has been found that the prevalence of IBS varies greatly from country to country. According to 53 studies that used the Roman III standard, the combined prevalence rate of IBS is 9.2% in 38 countries, and it is 3.8% in 34 countries shown by six studies that used the Rome IV standard (Oka et al., 2020). Although IBS is not a fatal disease, it actually reduces the quality of life of patients to a different extent.

The pathological mechanisms of IBS have not been discovered entirely, it may be related to gastrointestinal dysmotility, increased gut permeability, visceral hypersensitivity (VH), mucosal immune activation, intestinal dysbiosis, altered gut–brain interaction, and genetic and psychosocial factors (Gwee et al., 2019). Increasing evidence support that the dysfunction of microbiota–gut–brain axis is the important pathological basis of IBS (De Palma et al., 2014).

5-hydroxytryptamine (5-HT), also known as serotonin, is a key neurotransmitter and signal molecule in the brain–gut axis (Dy and Camilleri, 2000). About 90–95% of the 5-HT is produced in the gut, most of which is released by enterochromaffin cells (ECs). 5-HT exerts its biological effects by binding to various 5-HT receptors, among which 5-HT_3_ and 5-HT_4_ receptors are closely associated with the pathogenesis of IBS (Vahora et al., 2020). The reuptake of 5-HT is mainly performed by a serotonin transporter (SERT) to transport 5-HT into the cells, in which 5-HT is degraded and the metabolites are excreted from the body through the kidney (Bertrand and Bertrand, 2010). So, the number of ECs and the expression of SERT control the level of local 5-HT to a certain extent.

There is a complex bidirectional interaction between intestinal microbiota and 5-HT metabolism. Fung et al. (2019) found that increased 5-HT or fluoxetine (an inhibitor of SERT) in the intestinal tract can change the diversity of intestinal microorganisms and the colonization of some specific strains in mice. The studies of Jones et al. (2020) showed that intestinal microbiota and their metabolites affect the level of 5-HT in the gut and in the circulation by regulating the synthesis of 5-HT in ECs. Therefore, regulating intestinal microbiota is becoming an important way of treating IBS. And prebiotics, probiotics, antibiotics, dietary adjustment, and fecal microbiota transplantation (FMT) have been provided in clinical practice (Herndon et al., 2020).

FMT also known as “fecal transplantation” or “fecal bacteriotherapy,” is to transplant the functional bacteria derived from the feces of healthy people into the gastrointestinal tract of patients to restore the balance of intestinal microecology (Bakken et al., 2011). Pinn et al. (2014) reported that the symptoms were alleviated after FMT in 70% of patients with IBS who were not cured by conventional treatment. Huang et al. (2019) found that FMT can effectively relieve the symptoms of refractory patients with IBS, and the curative effect can be maintained for 3–6 months. Johnsen et al. (2018) deemed that the effect of frozen fecal microbiota is better than that of fresh fecal microbiota.

Shugan decoction (SGD) is a Chinese Herbal Prescription that can significantly ease IBS-D patients' abdominal pain, diarrhea, and emotional disorder by harmonizing liver–spleen according to the traditional Chinese medicine theory. Animal experiments showed that SGD can alleviate VH and defecation in water avoidance stress (WAS)-induced IBS model rats (Shang et al., 2013).

As mentioned above, the occurrence of IBS-D often accompanies disturbances of the intestinal microbiota. As a means of regulating the intestinal microbiota, FMT is an effective treatment for IBS-D. As a Chinese Herbal Prescription for treating IBS-D, SGD plays an important role in treating IBS-D. Both SGD and FMT have a therapeutic effect on IBS in human beings and animals, but the synergism between SGD and FMT has not been evaluated. Therefore, the aim of this study was to observe the effect of SGD combined with FMT on IBS-like symptoms induced by WAS in rats. In addition, we also explored whether FMT exerts its therapeutic effect on IBS rats *via* regulating the 5-HT signal in colon and evaluated the efficacy of filtered fecal microbiota liquid (FML).

## Materials and methods

### Agents and materials

SGD is composed of Bupleuri Radix (Chaihu) (Shanghai Hongqiao Traditional Chinese Medicine Co., Ltd. Lot number: 190806), Citri Reticulatae Pericarpium (Chenpi) (Shanghai Hongqiao Traditional Chinese Medicine Co., Ltd. Lot number: 190911), Paeoniae Radix Alba (Baishao) (Shanghai Hongqiao Traditional Chinese Medicine Co., Ltd. Lot number: 190213), Saposhnikoviae Radix (Fangfeng) (Shanghai Wanshicheng National Pharmaceutical products Co., Ltd. Lot number: 190921-1), Atractylodis Macrocephalae Rhizoma (Baizhu) (Shanghai Hongqiao Traditional Chinese Medicine Co., Ltd. Lot number: 190923), which were obtained from the pharmacy department of Longhua Hospital, Shanghai University of Traditional Chinese Medicine. Saikosaponin A (National Institute for Food and Drug Control, Lot number: 110777-201912), paeoniflorin (National Institute for Food and Drug Control, Lot number: 110736-201943), 5-O-methylvisammioside (National Institute for Food and Drug Control, Lot number: 111523-201811), hesperidin (National Institute for Food and Drug Control, Lot number: 110721-201818), and cimicifugoside (National Institute for Food and Drug Control; Lot number: 111522-201913) were purchased from Shanghai Chaorui Biological Technology Co., Ltd. (Shanghai, China). Antibodies used in this study include: Anti-Serotonin transporter antibody (Abcam: ab181034), GAPDH Mouse Monoclonal antibody (Proteintech, 6004-1-lg), Goat anti-Mouse IgG-HRP antibody (HUABIO, HA1006), Goat anti-Rabbit IgG-HRP antibody (HUABIO, HA1001), Donkey Anti-Rat IgG (Abcam, ab150155) and Goat Anti-Mouse IgG (Proteintech, SA00013-1).

### Preparation of SGD

The quality ratios of Bupleuri Radix (Chaihu), Citri Reticulatae Pericarpium (Chenpi), Paeoniae Radix Alba (Baishao), Saposhnikoviae Radix (Fangfeng), and Atractylodis Macrocephalae Rhizoma (Baizhu) are 6:3:4:4:6. SGD extract was prepared in the Herbal Chemistry Lab in Shanghai University of TCM. The extraction process is as follows: herbal pieces were soaked in distilled water for 30 min and then were boiled in six times of water for 1 h. The decoction was filtered by four layers of gauze and the residues were boiled again with 6-time water as above. The filtrates were mixed together, concentrated, and freeze-dried to powder.

### Analysis and identification of SGD by high-performance liquid chromatography

The ingredients of SGD were analyzed by HPLC as reported in our previous study (Wang et al., 2020). Briefly, Saikosaponin A, paeoniflorin, 5-O-methylvisammioside, hesperidin, or cimicifugoside was dissolved in methanol and obtained 1 mg/mL of standard solution separately. Five hundred milligrams of SGD extract power was weighed and dissolved in distilled water. After ultrasonic shock for 40 min, the SGD solution was fixed at a constant volume of 10 mL. One milliliter of solution was injected into the activated C_18_ column, and eluted with 10 mL water and 10 mL methanol in sequence. The methanol eluent was collected and was concentrated to dry, then dissolved in 1 mL of methanol, and a 50.89 mg/mL of SGD sample solution was obtained through filtrating by 0.45 μm microporous membrane. The standard solution and the SGD sample solution were analyzed using the Dionex UltiMate™ 3000 RSLC nano system (Thermo Scientific, MA, USA) equipped with a Corona^®^ultra™CAD detector, Luna^®^C18 Column (Phenomenex, 250 × 4.6 mm, 5 mm), and a data station with analytical software (CHROMELEON^®^). Mobile phases consisted of A-purified water and B-acetonitrile. Gradient was set as follows: 0 min, 5% B; 35 min 65.5% B; 35.001 min, 100% B; 40 min, 100% B. Column temperature was set at 25 °C, DAD detection wavelength: 203 nm, 254 nm, and 366 nm.

### Animals

Eighty-nine male Sprague-Dawley (SD) rats, weighing 200 g ± 20 g, were provided by Shanghai Bikai Experimental Animal Co., Ltd. (production license No.: SCXK [Shanghai] 2018-0006), and were raised in the Experimental Animal Center of Shanghai University of TCM under the standard temperature (21–24 °C), humidity (50% ± 5%), light and dark cycle (12 h/12 h), and they had free access to standard rat chow and tap water. All the experiments in this study are in accordance with the regulations of the Animal Ethics Committee of Shanghai University of TCM (No. PZSHUTCM190906001). All the experiments were carried out between 9:00 AM and 11:00 AM.

#### Preparation of FML

After a week of adaptive feeding, nine rats were randomly divided into three groups (*n* = 3 in each group): Control group: no treatment; WAS group: 10 days WAS; SGD group: 10 days WAS and dealing with SGD intragastric administration since the fourth day. Feces of rats in each group were collected. A certain quality of feces was weighed on a sterilized bending plate and was diluted with aseptic 0.9% NaCl solution at 37 °C with the ratio of 1: 10. FML was obtained after filtering by 2, 4, and 8 layers of aseptic gauze, respectively. FML filtration method: FML was centrifugated at 10,000 rpm and was filtrated with a 0.45 μm disposable needle filter. FMT was performed with 0.1 g feces per 100 g body weight.

#### Animals grouping and treatment

Experiment I: After a week of adaptive feeding, 56 rats were randomly divided into seven groups (*n* = 8 in each group): Control group: routine feeding; WAS→ Control group: routine feeding with FML1 (derived from rats in WAS group) gavage since the fourth day; WAS group: 10 days WAS on the base of routine feeding; SGD group: 10 days WAS with SGD gavage since the fourth day on the base of routine feeding; Control→ WAS group: 10 days WAS with FML2 (derived from rats in Control group) gavage since the fourth day on the base of routine feeding; SGD→ WAS group: 10 days WAS FML3 (derived from rats in SGD group) gavage since the fourth day on the base of routine feeding; SGD + (Control→ WAS) group: 10 days WAS with SGD and FML2 (derived from rats in Control group) gavage since the fourth day on the base of routine feeding.

Experiment II: After a week of adaptive feeding, 24 rats were randomly divided into three groups (*n* = 8 in each group). Control group: routine feeding; Control→ WAS group: 10 days WAS with FML2 (derived from rats in Control group) gavage since the fourth day on the base of routine feeding; FControl→ WAS group: 10 days WAS with FML2 (derived from rats in Control group) filtrate gavage since the fourth day.

### Water avoidance stress

Refer to the method initiated by (Bradesi et al., 2005; Wang et al., 2020), the rats were placed on a platform (10 cm long, 8 cm wide, 8 cm high) which was fixed in the center of an organic glass pool (45 cm long, 25 cm wide, 25 cm high) filled with water (25 °C) to suffer from WAS for 1 h every day in 10 consecutive days.

### Fecal pellets counting

The amount of FPT of rats during WAS was counted every day.

### Colorectal distension

On the 10^th^ day after WAS, the pressure threshold to induce abdominal withdrawal reflex (AWR) in rats was measured by colorectal distension (CRD) test. The methods were as follows: a balloon (5 mm diameter and 1 cm long) with a catheter (2 mm diameter) was inserted into the colorectum 1 cm above the anus. The catheter was fixed to the root of the rat tail with adhesive tape. Then the balloon was inflated gradually by one experimenter and the pressure values were monitored. Meanwhile, the abdominal wall reactions of the rats were observed by the other experimenter and a voice sign was made by him when the first AWR appeared and the immediate pressure value was recorded by the former experimenter. The average value, which was named the pain pressure threshold (PPT), was calculated after three repeated measurements which were performed with a 3-min interval between every two successive measurements.

### HE stain and immunofluorescence

The colon tissue was fixed in 4% paraformaldehyde for 48 h after the content was washed off with ice normal saline. Then the paraffin sections were made by dehydration, transparency, wax soaking, embedding, and sectioning. Hematoxylin-eosin (HE) solution staining, neutral gum sealing, and observation under the ordinary optical microscope (Nikon Corporation, Japan) were done in sequence. The paraffin slice was put into buffering solution of citric acid to repair the antigen for 15 min in a microwave oven after dewaxing. Then it was washed with PBS three times (5 min each time) after cooling. The tissue was incubated with 3% H_2_O_2_ for 15 min and was washed before it was incubated with serotonin antibody (1:250) and Chromogranin A (1:100) antibody overnight at 4 °C. Then it was incubated with secondary antibodies (donkey anti-rat IgG 1:500 and Goat Anti-Mouse IgG 1:250) after it was washed with PBS three times (5 min each time). LSM800 laser confocal microscope (Zeiss, Germany) was used to observe the immunofluorescence after adding anti-quenching DAPI and seal.

### Protein extraction and western blotting

The shredded colonic tissue was put into the RIPA lysis buffer with a protease inhibitor for homogenate. And then the homogenate was centrifuged at 4 °C, 12,000 rpm for 15 min, and the supernatant was collected. BCA protein assay kit (CWBIO: CW0014s) was used to measure protein concentration. Samples were mixed with 5 × loading buffer and were heated at 95 °C for 10 min to denature. Then, 100 mg of total proteins were loaded on 10% SDS polyacrylamide gels and electrophoresed. The proteins were then transferred to the PVDF membrane (Millipore, Darmstadt, Germany). The PVDF membrane was incubated with 5% BSA for 1 h and then was incubated with SERT antibody (1:1,000) overnight. Then it was incubated with a secondary antibody (goat anti-rabbit IgG-HRP1:20,000) for 1 h after it was washed with Tris-buffered saline and Tween 20 (TBST) three times (10 min each time). The membranes were washed again. Specific protein bands were visualized using the ECL kit (Millipore: WBKLS500) and imaged with SyngeneG (BOX ChemiXT4).

### Statistical analysis

SPSS version 24.0 (SPSS, Chicago, IL, USA) and GraphPad Prism 5.0 (La Jolla, CA, USA) were used for data analysis. Each value was expressed as mean ± SE. If data were subject to normality and homogeneity of variance, an one-way analysis of variance (one-way ANOVA) and followed LSD *t*-test was used for analyzing the differences among the groups. If disobedient, the rank-sum test was used. *P* < 0.05 was considered statistically significant.

## Results

### Chemical composition of SGD

**Figure 1:**
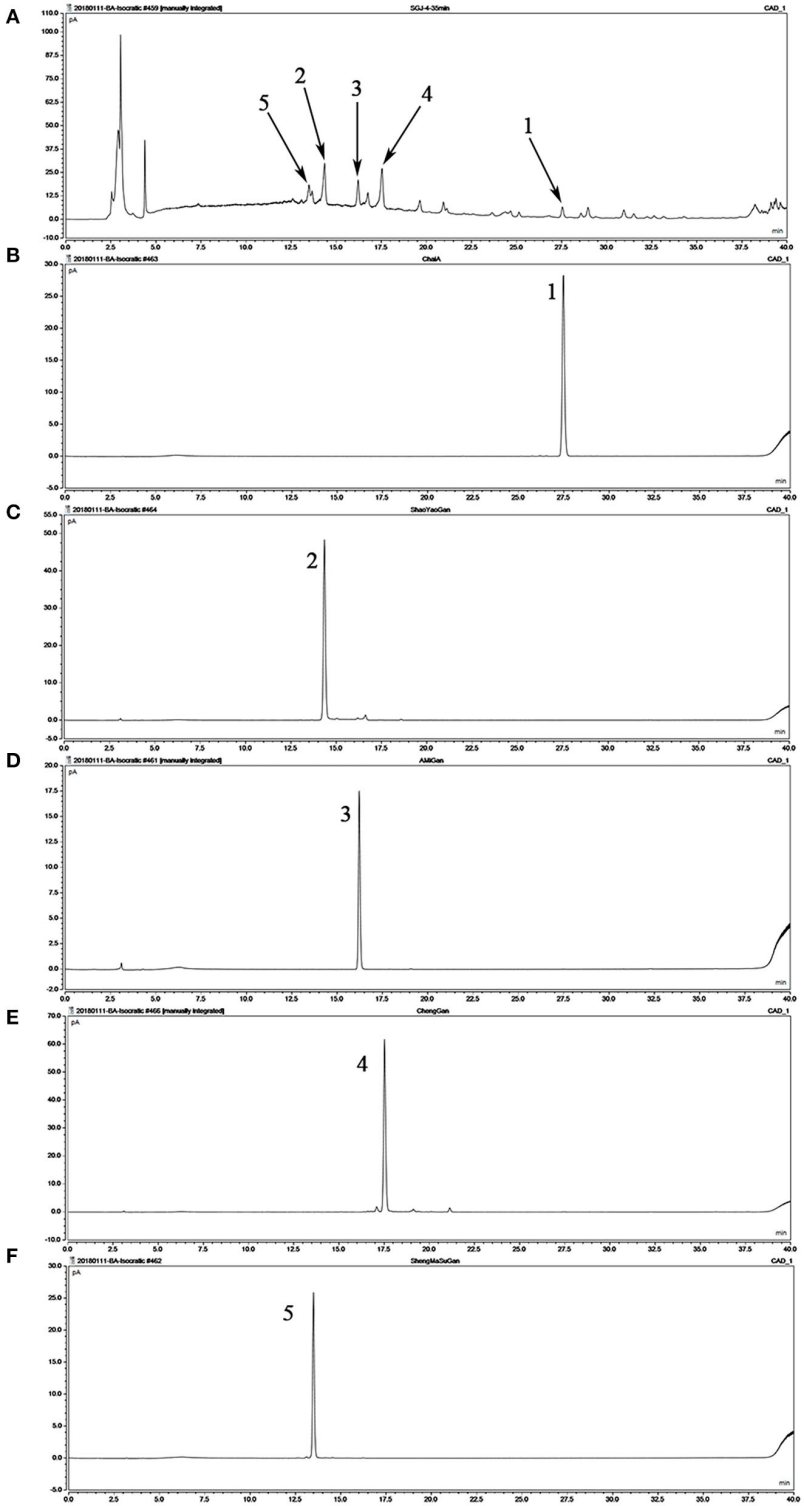
Analysis of the chemical composition of Shugan decoction (SGD) extract by HPLC. **(A)** HPLC chromatogram of SGD extract; **(B)** HPLC chromatogram of saikosaponin A; **(C)** HPLC chromatogram of paeoniflorin; **(D)** HPLC chromatogram of 5-O-methylvisammioside; **(E)** HPLC chromatogram of hesperidin; **(F)** HPLC chromatogram of cimicifugoside. Peak 1: saikosaponin A; Peak 2: paeoniflorin; Peak 3: 5-O-methylvisammioside; Peak 4: hesperidin; Peak 5: cimicifugoside.

Referring to the components of SGD in Chinese Pharmacopeia, we confirmed the principal ingredients of SGD extract as follows: saikosaponin A, paeoniflorin, 5-O-methylvisammioside, hesperidin, and cimicifugoside (Figure 1).

### Effect of FML1 on normal rats

**Figure 2:**
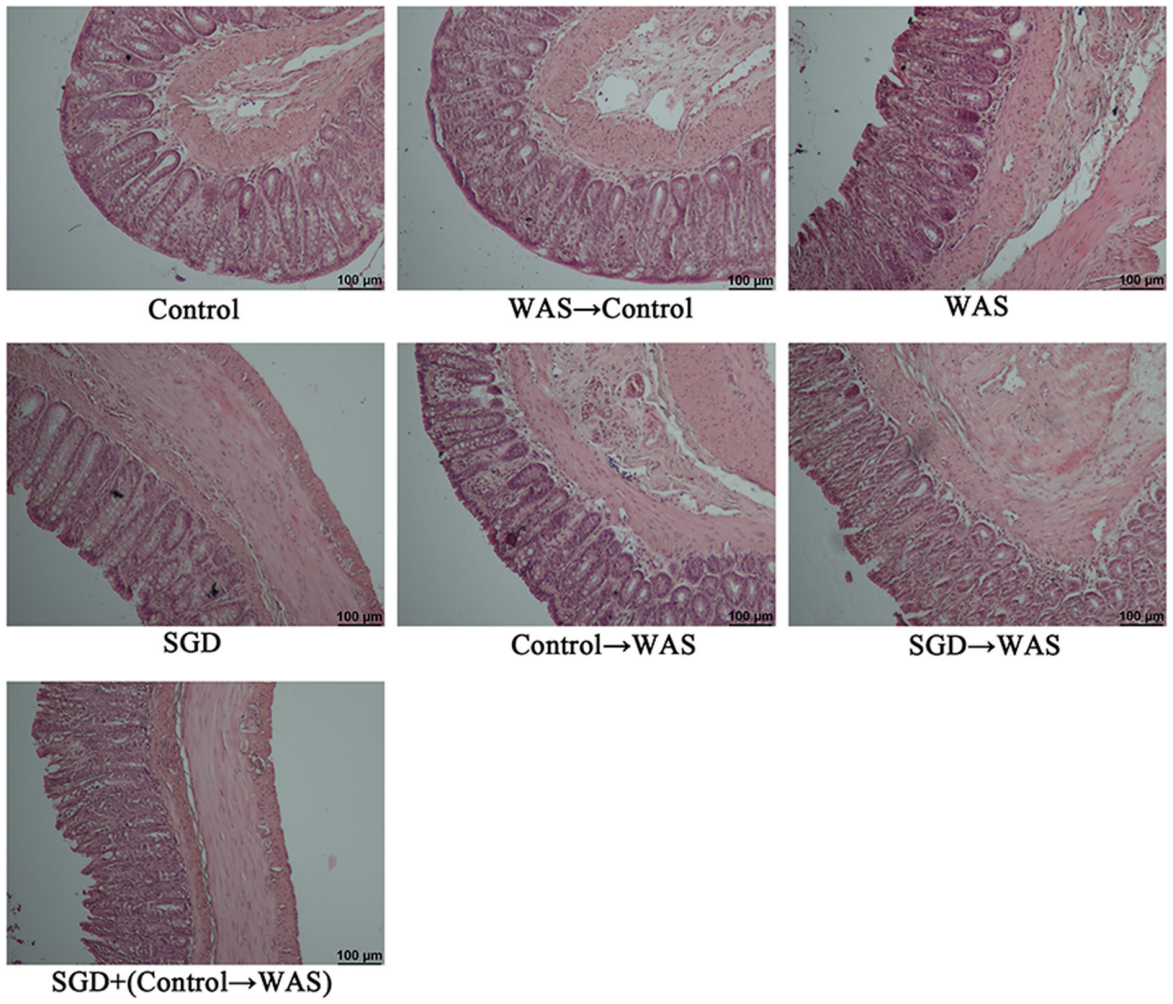
Histopathological examination of colon in rats.

HE staining showed that FML1-like WAS did not induce colonic pathological changes in normal rats (Figure 2).

**Figure 3:**
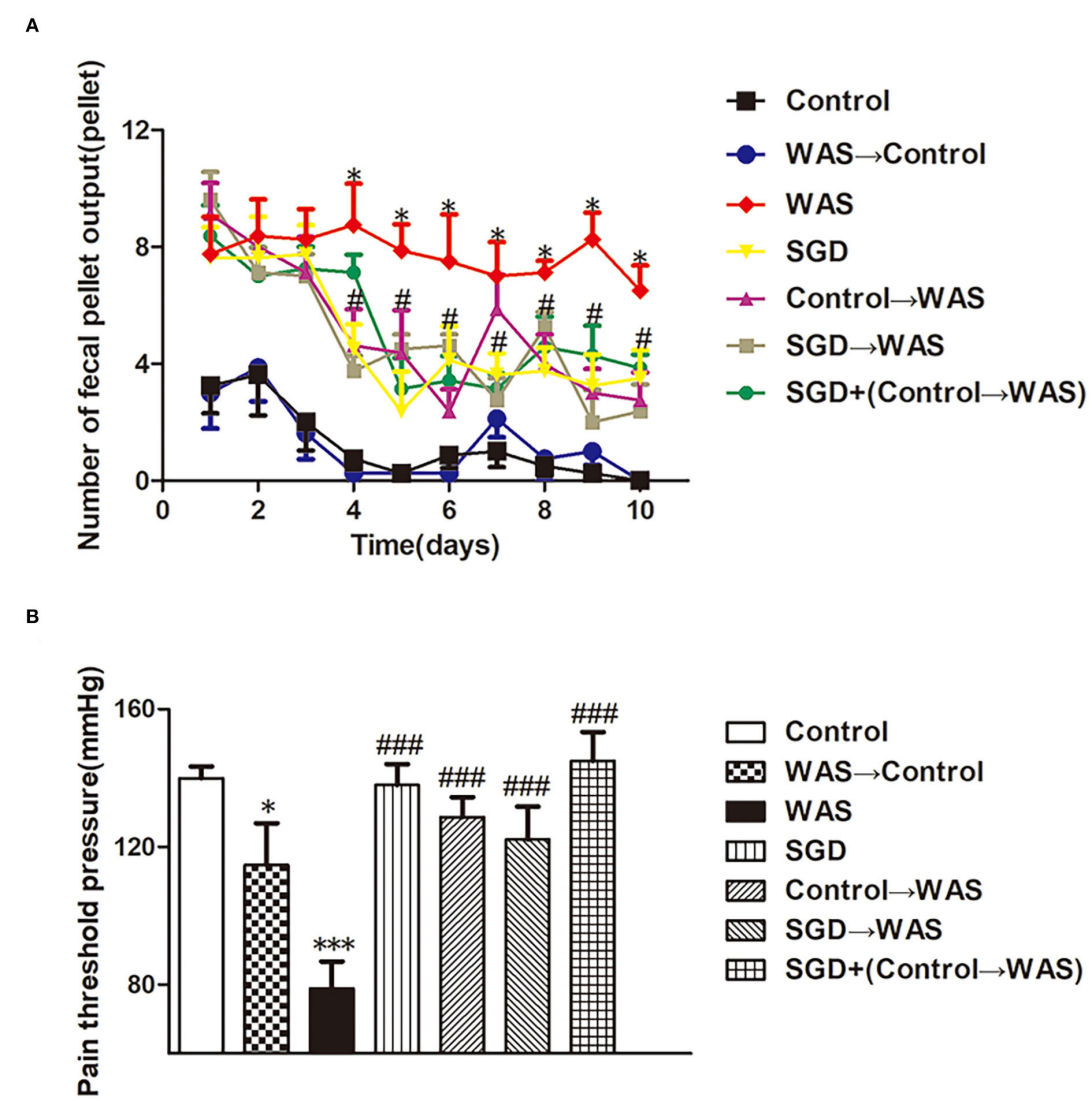
Effects of several FMT combined with or without SGD on IBS-like symptoms of WAS rats. **(A)** Fecal pellet numbers output of rats every day. **(B)** PPT in rats (All results are expressed as mean ± SE *n* = 8/group, *P < 0.05 vs. Control, ***P < 0.001 vs. Control, ^#^*P* < 0.05 vs. WAS, ^###^*P* < 0.001 vs. WAS).

Compared with that in the control group, the PPT in the WAS→ Control group, as well as that in the WAS group, decreased significantly (*P* < 0.05); but the number of FPT of rats in the WAS→ Control group was not increased (*P* > 0.05) although that in the WAS group increased significantly (*P* < 0.05) (Figure 3).

### Effect of SGD combined with FML2 on WAS rats

HE staining showed that FML2-like SGD did not influence the colon mucosa obviously (Figure 2).

Compared with that in WAS group, the number of FPT of rats in the SGD group, Control→ WAS group, SGD→ WAS group, or SGD + (Control→ WAS) group was significantly reduced (*P* < 0.05, *P* < 0.05, *P* < 0.05, *P* < 0.05), accompanied with the significantly increased PPT (*P* < 0.001, *P* < 0.001, *P* < 0.001, *P* < 0.001), but there was no significant difference among that in these four groups (*P* > 0.05) (Figure 3).

### Effect of FML2 filtrate on WAS rats

**Figure 4:**
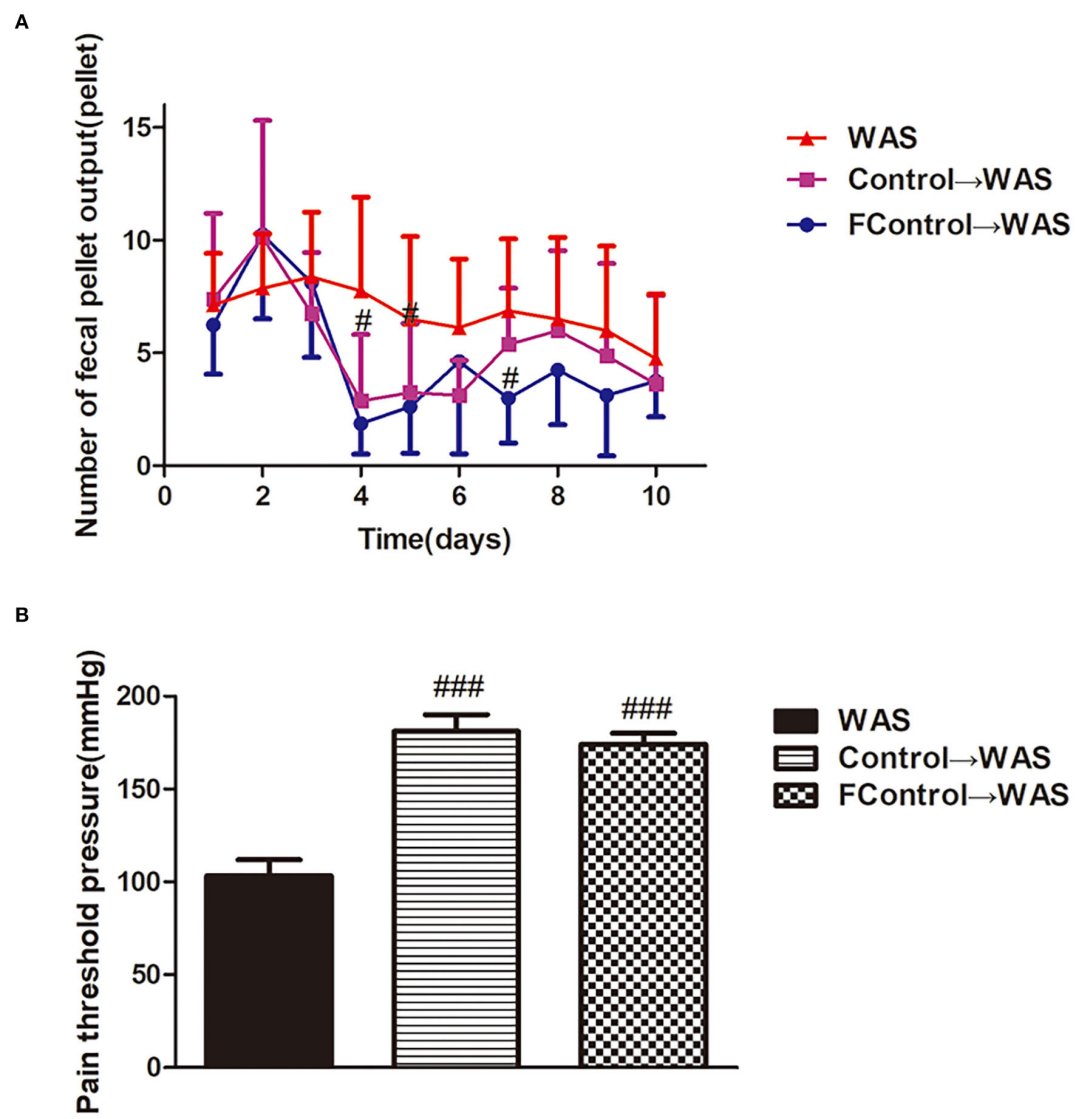
Effect of filtered FML on IBS-like symptoms of WAS rats. **(A)** Fecal pellet numbers output of rats every day. **(B)** PT in rats (All results are expressed as mean ± SE *n* = 8/group, ^#^*P* < 0.05 vs. WAS, ^###^*P* < 0.001 vs. WAS).

We compared the effects of filtered and unfiltered FML2 on the abnormal colonic motility and VH of WAS rats by transplantation to further confirm whether the microbiota or their metabolites in FML2 played the primary role. It was shown that the number of FPT of rats in either the Control→ WAS group or FControl→ WAS group was significantly reduced on the fourth and fifth day (*P* < 0.05, *P* < 0.05) with the increased PPT (*P* < 0.001, *P* < 0.001) compared to that in WAS group. But there were no significant differences between that in the Control→ WAS group and that in the FControl→ WAS group (*P* >0.05) (Figure 4).

### Effect of FML1 on 5-HT content, ECs number, and SERT expression in the colon of normal rats

**Figure 5:**
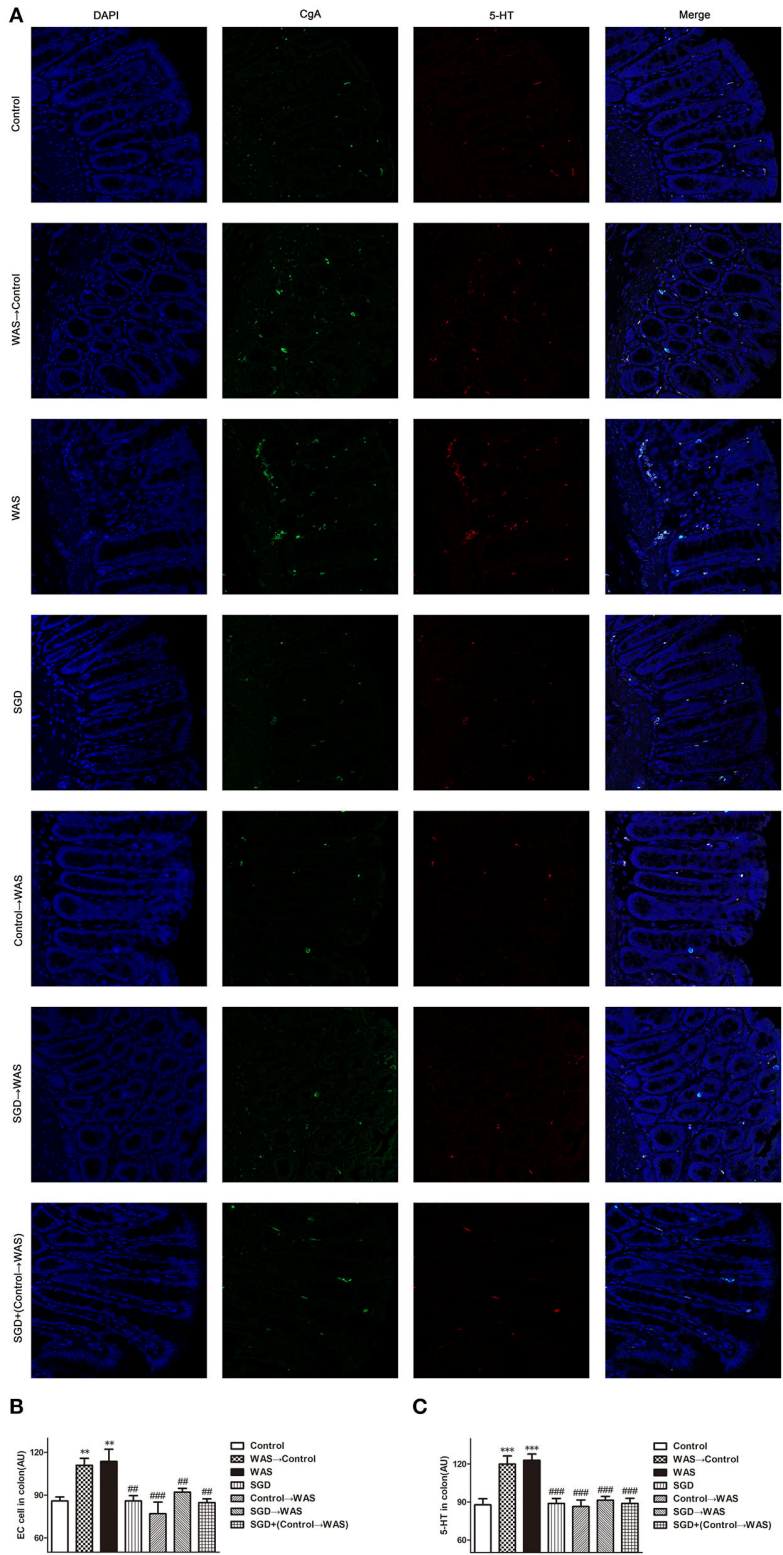
Effects of several FMT combined with or without SGD on ECs numbers and 5-HT content in the colon of rats. **(A)** Colon immunofluorescence staining in rats. **(B)** Statistical plot of ECs numbers in the colon. **(C)** Statistical plot of the 5-HT content in the colon (All results are expressed as mean ± SE *n* = 5/group, **P < 0.01 vs. Control, ***P < 0.001 vs. Control, ^##^*P* < 0.01 vs. WAS, ^###^*P* < 0.001 vs. WAS).

**Figure 6:**
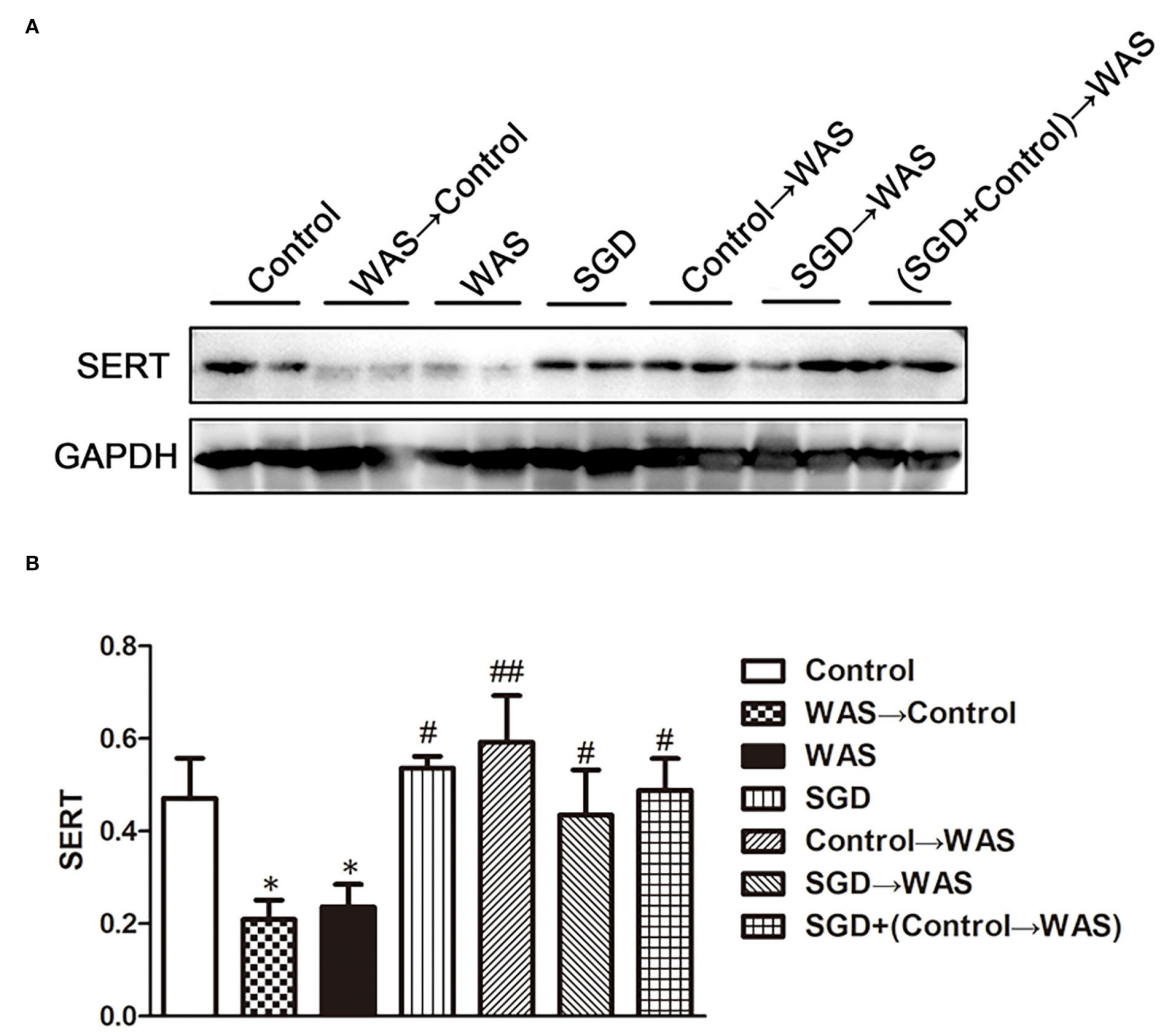
Effects of several FMT combined with or without SGD on SERT expression in the colon of rats. **(A)** SERT protein expression plot in the colon. **(B)** Statistical plot of SERT protein expression in the colon (All results are expressed as mean ± SE n = 3/group, *P < 0.05 vs. Control, ^#^*P* < 0.05 vs. WAS, ^##^*P* < 0.01 vs. WAS).

Compared with that in Control group, the intensity of green (ECs) and red (5-HT) fluorescence in the colon of rats in WAS→ Control group was increased significantly (*P* < 0.01, *P* < 0.001) (Figure 5), while the expression of SERT protein was decreased significantly (*P* < 0.05) (Figure 6).

### Effect of FML2 on 5-HT content, ECs number, and SERT expression in the colon of WAS rats

Compared with that in the WAS group, the intensity of green (ECs) and red (5-HT) fluorescence in the colon of rats in the Control→ WAS group or in the SGD→ WAS group was decreased significantly (*P* < 0.01, *P* < 0.001) (Figure 5), and the expression of SERT protein was increased significantly (*P* < 0.05, *P* < 0.01) (Figure 6).

### Effect of filtered FML on 5-HT content, ECs number, and SERT expression in the colon of WAS rats

**Figure 7:**
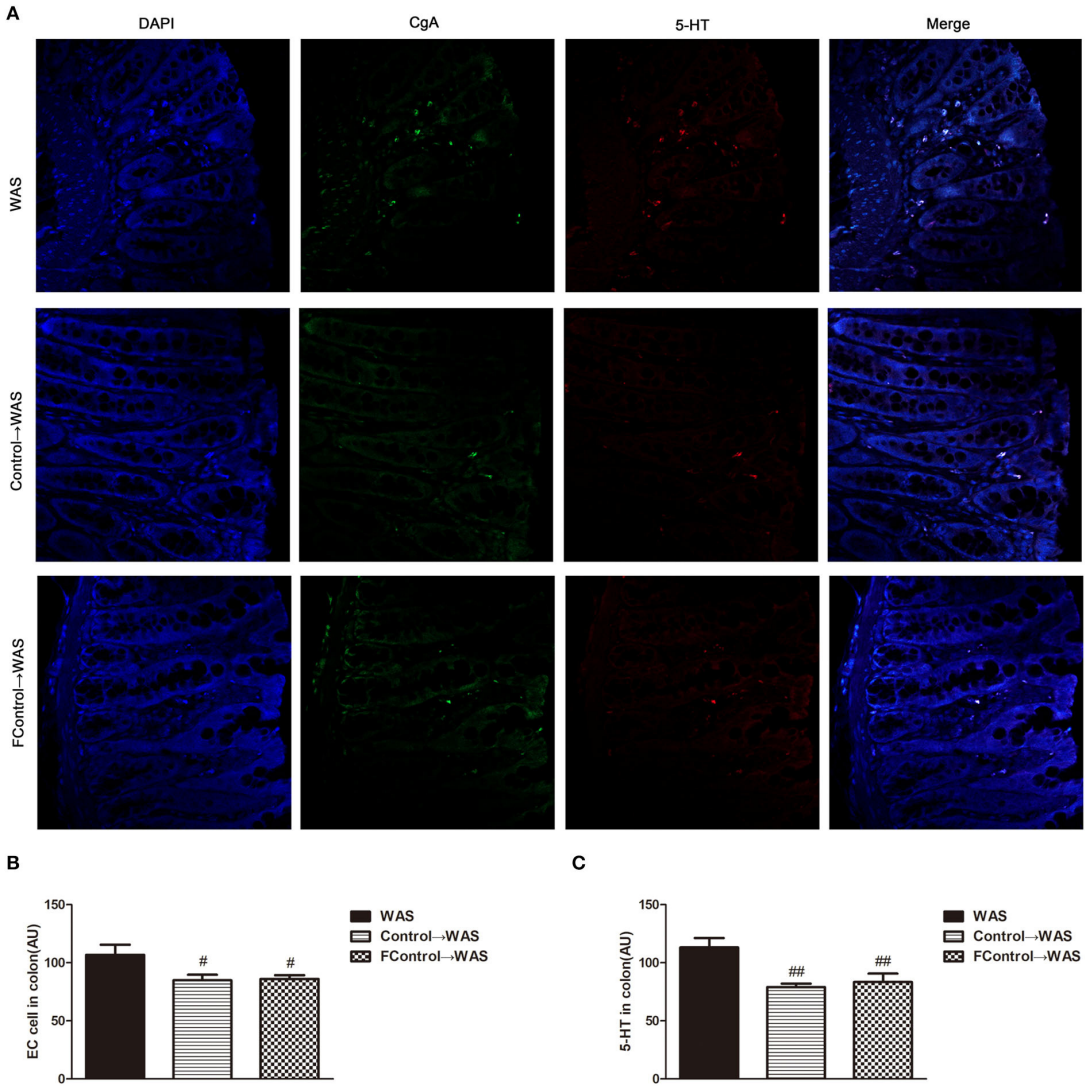
Effect of filtered FML on ECs numbers and 5-HT Content in the Colon of Rats. **(A)** Colon immunofluorescence staining in rats. **(B**) Statistical plot of ECs numbers in the colon. **(C)** Statistical plot of the 5-HT content in the colon (All results are expressed as mean ± SE n = 5/group, ^#^*P* < 0.05 vs. WAS, ^##^*P* < 0.01 vs. WAS).

**Figure 8:**
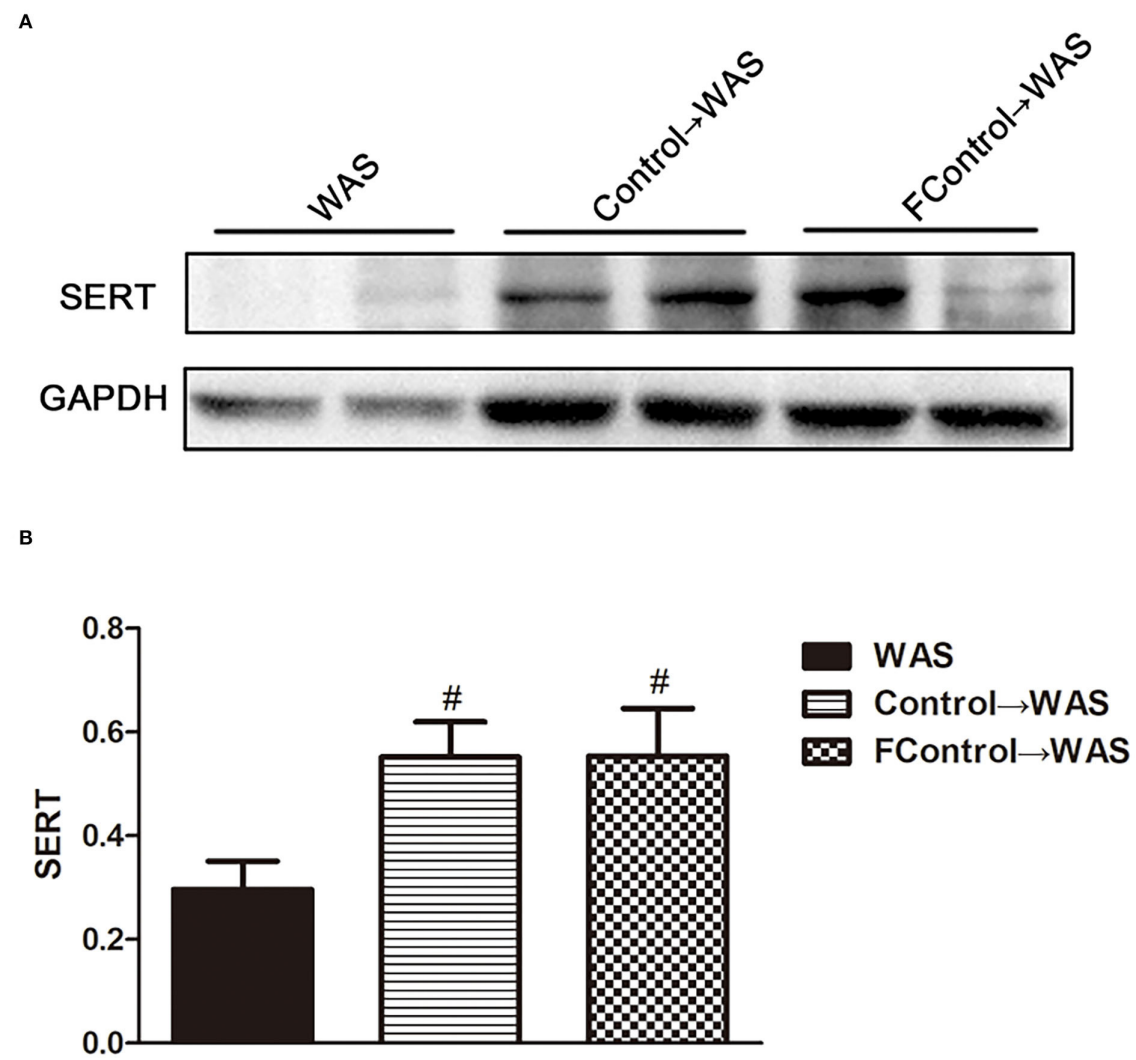
Effect of filtered FML on SERT expression in the colon of rats. **(A)** SERT protein expression plot in the colon. **(B)** Statistical plot of SERT protein expression in the colon (All results are expressed as mean ± SE n = 3/group, ^#^*P* < 0.05 vs. WAS).

Compared with that in WAS group, the intensity of green (ECs) and red (5-HT) fluorescence in the colon of rats in the FControl→ WAS group was significantly reduced (*P* < 0.05, *P* < 0.01) (Figure 7), and the expression of SERT protein was increased significantly (*P* < 0.05) (Figure 8).

## Discussion

Intestinal microecological imbalance plays an important role in the pathogenesis of IBS. It is suggested that neurotransmitters, compounds, metabolites, enzymes, and endocrine factors derived from intestinal microbiota may be involved in the pathogenesis of IBS (Mishima and Ishihara, 2020), namely, abnormal intestinal motility, VH, damaged intestinal mucosal barrier, and wrong neuro-immune signals, which are inextricably linked to the intestinal microbiota (Distrutti et al., 2016). In this study, we found that transplanting FML1 (derived from WAS rats) into control rats increased the visceral sensitivity of control rats, which maybe indirectly support WAS inducing VH by damaging gut microecological balance. However, FML1 transplantation did not influence the number of FPT of control rats, which suggested that the mechanisms involved in WAS-induced rat IBS are complex and are not limited to intestinal dysbiosis. SGD gavage, transplanting FML2 (derived from control rats), or transplanting FML3 (derived from rats in the SGD group) could reduce the number of FPT and decrease the visceral sensitivity of WAS rats. But it failed to show the synergistic effect of SGD and FML2 transplantation, which may be due to SGD and FML2 sharing the same way-regulating intestinal microbiota to improve VH and abnormal defecation induced by WAS in rats. This finding indicates that although either SGD or FMT is effective, the combination therapy of them is unnecessary in the clinical practice of IBS treatment.

The intestinal microbiota is involved in the regulation of metabolism of 5-HT. The results of this study confirmed that transplanting FML1 to normal rats could increase the content of 5-HT in colon. The simultaneously increased number of ECs and decreased expression of SERT may contribute to the elevated 5-HT level. Interestingly, there are other studies that reported transplanting the intestinal microbiota from normal mice to germ-free mice could cause a significant increase of 5-HT levels in colon of the latter (Yano et al., 2015; Hata et al., 2017; Yang et al., 2017), which seems to be quite contrary to our result. After all, germ-free mice are different from normal mice. SGD gavage, transplanting FML2, or transplanting FML3 could decrease the high level of 5-HT in colon of WAS rats by normalizing the number of ECs and the expression of SERT in colon maybe via regulating gut microbiota. Cao et al. (2018) have shown that the supernatant of Lactobacillus acidophilus and *Bifidobacterium longum* could upregulate the mRNA and protein levels of SERT in intestinal epithelial cells. We did not know what component of FML derived from control rats, i.e., FML2, exerted their regulating effect on IBS symptoms, 5-HT level, ECs number, and SERT expression in colon of WAS rats yet.

To further identify microbiota or their metabolites in FML2 play the primary role in alleviating the IBS-like symptoms of WAS rats, we compared the effect of filtered FML2 (generally, it is regarded that bacteria can be eliminated by a 0.45 μm filter) and unfiltered FML2 on the IBS-like symptoms of WAS rats. It was found that filtered FML2 and unfiltered FML2 are equally effective. We speculated that the metabolites of the microbiota seem to play a major role in FMT. Certainly, this speculation needs to be confirmed further. In fact, a similar report has shown that filtered fecal solution FMT could treat *Clostridium difficile* infections (Ott et al., 2017). Recent studies suggested that changes in metabolites of intestinal microbiota such as short-chain fatty acids (SCFAs) and bile acids may be involved in the pathogenesis of IBS. A meta-analysis on the levels of SCFAs in feces from healthy people and from patients with IBS suggested that butyrate in feces from patients with IBS-D is significantly increased and that propionate and butyrate in feces from patients with IBS-C are significantly decreased compared with that in feces from healthy people (Sun et al., 2019). The significantly increased concentration of SCFAs in feces of IBS-D model mice has also been reported (Shaidullov et al., 2021). It has been realized that changes in bile acid metabolism are associated with diarrhea and VH in patients with IBS (Wei et al., 2020). Li et al. (2019) have shown that bile acid induces VH through signal transduction of mucosal mast cells to pain receptors.

In summary, our data suggested that both SGD and FMT with healthy FML can effectively improve IBS-like symptoms and regulate colon 5-HT levels in WAS rats, but they have no synergistic effect. Therefore, the combination of FMT and traditional Chinese medicine compounds like SGD is not clinically recommended for IBS. The metabolites of intestinal microbiota may play an important role as effective substances in the treatment of FMT on IBS. Therefore, when using FMT for IBS, we recommend filtered FMT, which can effectively reduce infection risk due to microbiota invasion (Ott et al., 2017). Nevertheless, the animal model could not fully reflect the pathophysiology of human disease, and the therapeutic effect of FMT combined with or without SGD on patients with IBS needs to be further verified.

## Data availability statement

The original contributions presented in the study are included in the article/supplementary material, further inquiries can be directed to the corresponding author.

## Ethics statement

The animal study was reviewed and approved by the Animal Ethics Committee of Shanghai University of TCM.

## Author contributions

YM and YF wrote the draft of the paper. LH, YZ, EW, YM, and YF carried out the animal experiments. JY designed the experiments and revised the paper. All authors contributed to the article and approved the submitted version.

## Funding

This work was supported by the National Natural Science Foundation of China (No. 81874391).

## Conflict of interest

The authors declare that the research was conducted in the absence of any commercial or financial relationships that could be construed as a potential conflict of interest.

## Publisher's note

All claims expressed in this article are solely those of the authors and do not necessarily represent those of their affiliated organizations, or those of the publisher, the editors and the reviewers. Any product that may be evaluated in this article, or claim that may be made by its manufacturer, is not guaranteed or endorsed by the publisher.
